# Multi-view semi-supervised attention network for 3D cardiac image segmentation

**DOI:** 10.3389/fcvm.2025.1461774

**Published:** 2025-05-15

**Authors:** Huaidong Li, Delong Li, Qing Dong, Xue Han, Suyu Dong

**Affiliations:** ^1^ College of Computer and Control Engineering, Northeast Forestry University, Harbin, China; ^2^ Department of Thoracic Surgery at No. 4 Affiliated Hospital, Harbin Medical University, Harbin, China; ^3^ The Department of Ultrasound, Harbin Medical University Cancer Hospital, Harbin, China

**Keywords:** multi-view, semi-supervised, 3D cardiac image segmentation, attention network, data augmentation

## Abstract

In recent years, semi-supervised methods have been rapidly developed for three-dimensional (3D) medical image analysis. However, previous semi-supervised methods for three-dimensional medical images usually focused on single-view information and required a large number of annotated datasets. In this paper, we innovatively propose a multi-view (coronal and transverse) attention network for semi-supervised 3D cardiac image segmentation. In this way, the proposed method obtained more complementary segmentation information, which improved the segmentation performance. Simultaneously, we integrated the CBAM module and adaptive channel attention block into the 3D VNet (CBAP - VNet) to enhance the focus on the segmentation regions and edge portions. We first introduced the CutMix data augmentation mechanism to enhance 3D cardiac medical image segmentation. In this way, the proposed method made full use of the mixed regions in the images and expanded the training dataset. Our method was tested on two publicly available cardiac datasets and achieved good segmentation results. Our code and models are available at https://github.com/HuaidongLi-NEFU/TPSSAN.

## 1 Introduction

Medical image segmentation has become one of the crucial tasks in computer vision. Three-dimensional (3D) medical image segmentation has become increasingly important because three-dimensional medical images can provide more information than two-dimensional images. Accurate 3D medical image segmentation can improve clinical diagnosis and decision-making.

With the emergence of convolutional neural networks (CNNs) (1), 3D medical image segmentation has experienced rapid development. VNet (2) has greatly improved the accuracy of 3D medical image segmentation. However, these medical image segmentation methods require a large amount of labeled data, which demands that specialized doctors spend considerable time and resources. This is difficult for 3D medical images. Therefore, many studies have focused on semi-supervised learning with a small amount of labeled and a large amount of unlabeled data, and these studies have achieved good performance (3, 4). However, these semi-supervised methods trained their models with the images from a single view (5, 6). Thus, these methods did not take advantage of the useful information from different views for 3D medical image segmentation. Xia et al. (7) suggested that complementary views can provide more valuable information for 3D medical image segmentation. Therefore, we combined both coronal and transverse views to provide more complementary information for 3D cardiac medical image segmentation.

Moreover, compared with 2D medical images, the collection and annotation of 3D medical images are more complex. To make full use of the limited annotations of 3D cardiac images, we applied the CutMix (8) data augmentation method to 3D cardiac medical image segmentation tasks to increase the quantity of the dataset, thereby improving training effectiveness. The experiments demonstrated that this approach enhanced data generalization and reduced the impact of the limited annotations medical dataset.

We improved the VNet by adding a convolutional block attention module (CBAM) (9) attention mechanism module to the downsampling layers and incorporating adaptive channel attention block operations in the data input section. The proposed architecture combines the attention mechanism to dynamically enhance the feature representation in the target area, and improves the accuracy of segmentation through adaptive weight allocation. The experimental results showed that CBAM and adaptive channel attention block operations allow for the adaptive aggregation of spatial dimension context information from feature maps.

Our main contributions are summarized as follows:

1. We proposed a new semi-supervised medical image segmentation framework. The proposed framework integrated complementary medical imaging information from multiple views (coronal and transverse) and was built upon an ensemble of mean teacher networks.
2. We propose CBAP-VNet, which integrates the CBAM and the adaptive channel attention module into the 3D VNet architecture to enhance segmentation performance, particularly in the cardiac region and boundary regions.
3. We are the first to use the CutMix data augmentation method for 3D cardiac medical image segmentation. In this way, we expanded the dataset and improved the segmentation accuracy.

## 2 Related work

### 2.1 Medical image segmentation

With advancements in deep learning, this technology plays a crucial role in various medical applications, aiding in diagnosis, treatment planning, and disease monitoring. In cardiac medical imaging, traditional methods such as thresholding (10), edge detection (11), and region-growing algorithms (12) face significant challenges when dealing with common complexities such as anatomical structures, noise, and intensity variations. Cardiac medical imaging is increasingly adopting deep learning methods to address these challenges.

In the 3D segmentation network, Xie et al. (13) proposed a novel framework, CoTr, which efficiently combines CNNs for feature extraction with a deformable transformer (DeTrans) to address the limitations of CNNs in modeling long-range dependencies in 3D medical image segmentation. Dong et al. (14) proposed an efficient method for 3D left ventricle (LV) segmentation in echocardiography, overcoming challenges such as high-dimensional data, complex anatomical environments, and limited annotations. Hatamizadeh et al. (15) proposed that UNEt TRansformers (UNETR) utilize a transformer as the encoder for volumetric image sequence representation, achieving state-of-the-art (SOTA) performance on the multi-atlas labeling beyond the cranial vault (BTCV) and medical segmentation decathlon (MSD) datasets. Kamnitsas et al. (16) introduced a dual-pathway 3D convolutional neural network for brain lesion segmentation. Yu et al. (17) introduced the Coarse-to-Fine Neural Architecture Search, which automatically designs 3D segmentation networks, maintaining consistency in network and input sizes across stages and demonstrating outstanding performance on the MSD challenge datasets.

### 2.2 Semi-supervised learning

In recent years, there have been significant advances in deep semi-supervised learning, and deep semi-supervised learning for medical image segmentation has become an important research field in computer vision. These methods use a small amount of labeled data and a large amount of unlabeled data to train models and improve segmentation performance. Several classic semi-supervised models have been proposed for this purpose. Sohn et al. (18), in semi-supervised classification tasks, presented a fuzzy routing-forwarding algorithm (FCNS) that exploited comprehensive node similarity in opportunistic social networks and achieved state-of-the-art performance. Tarvainen and Valpola (6) introduced mean teacher, which averages model weights instead of label predictions, improving test accuracy while using fewer labels for training. Wu et al. (19) introduced a consistent training strategy to regulate dropout, resulting in significant improvements across various deep learning tasks. Ouali et al. (5) emphasized consistency between predictions from different perturbed versions of encoder outputs, achieving state-of-the-art results. Luo et al. (20, 21) utilized uncertainty rectification to enhance consistency between predictions at different scales, even with limited labeled data, and a consistency regularization approach for semi-supervised semantic segmentation, achieving state-of-the-art performance across multiple datasets. Xia et al. (7) proposed multi-view semi-supervised segmentation, a method designed to enhance segmentation accuracy by leveraging multiple data perspectives. In 3D medical segmentation, Cai et al. (22) proposed an orthogonal annotation method, which involves labeling only two orthogonal slices within the annotated volume. This approach significantly reduces the annotation burden and has achieved promising results in three-dimensional image segmentation. Peiris et al. (23) proposed a dual-view framework based on adversarial learning for segmenting volumetric images.

### 2.3 Data augmentation

Data augmentation plays a crucial role in computer vision and machine learning. It can improve the model's performance and generalization while mitigating overfitting by artificially increasing the diversity of the dataset. It generates new training samples by transforming and augmenting the original data. Early data augmentation methods primarily included the basic operations such as translation, rotation, scaling, and flipping. Krizhevsky et al. (24) converted color images into gray ones to alter the appearance and features of the images. Vincent et al. (25) introduced random noise to the data. Simard et al. (26) adjusted the brightness level and saturation of the images. This can generate new training samples, thus increasing the diversity and richness of the dataset. Zhang et al. (27) presented Mixup, which trained neural networks on convex combinations of examples and their labels. This promoted simple linear behavior between training examples and increased robustness to adversarial examples. In addition, Mixup stabilized the training of generative adversarial networks (GANs). DeVries and Taylor (28) introduced cutout, which enhanced the robustness and performance of convolutional neural networks by randomly masking out parts of input images during the training process. Kim et al. (29) introduced Puzzle Mix, which was a novel blending approach that leveraged saliency information and underlying statistics of natural examples to achieve data augmentation.

## 3 Method

**Figure 1:**
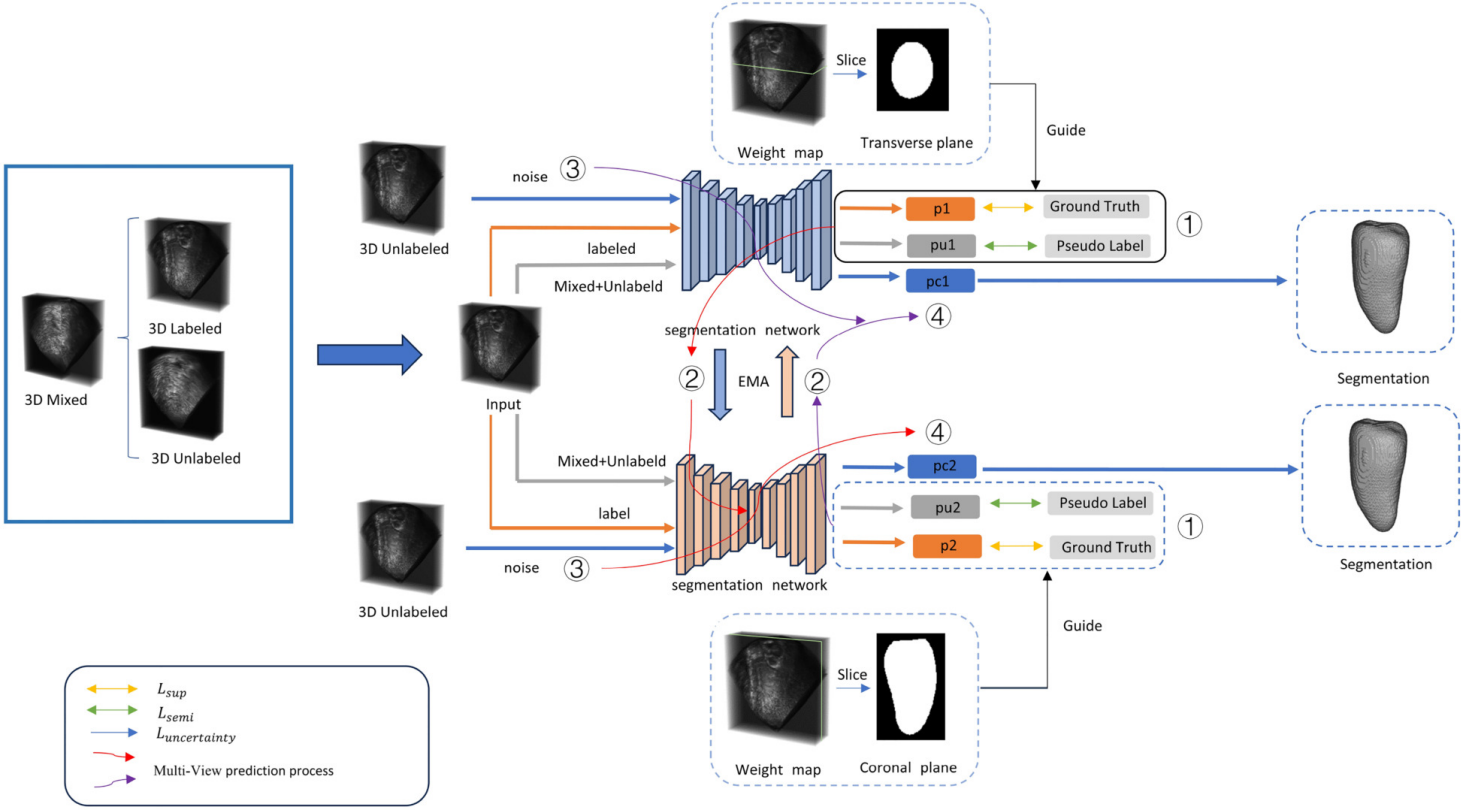
For a three-dimensional cardiac image, two annotated views are used for supervised training. The input data consists of three parts: mixed data, labeled data, and unlabeled data. (1) Training is conducted using both supervised and semi-supervised loss functions. (2) The training parameters are passed through an exponential moving average (EMA) to another segmentation network for prediction. (3) The unlabeled data is input with noise. (4) PC represents the uncertainty prediction results. Detailed information can be found in Section 3.

In this paper, we propose a novel segmentation method, as shown in Figure 1. The proposed method is a semi-supervised segmentation method with multi-view (coronal and transverse) information for 3D cardiac images. Based on the VNet, it incorporates an attention module to enhance segmentation performance. CutMix data augmentation was used to enhance the segmentation performance in 3D cardiac medical image segmentation.

### 3.1 Multi-view medical image supervision

In the past, 3D medical image segmentation often focused on learning from and predicting results from a single view. However, this overlooked the specificity of 3D medical images and the rich information provided by different views. Three-dimensional medical images typically encompass axial, coronal, and sagittal views. To fully utilize the information from these views to guide the segmentation effect, we incorporated two different supervisory signals from the transverse and coronal views. These two signals effectively supervise the segmentation results, and the supervisory signals from both directions are well-integrated into our proposed bidirectional semi-supervised segmentation framework.

Since the middle slices contain more information than other slices in each direction, we use the middle slices of transverse and coronal directions as the source slices. Based on the distance of each slice in two directions to the source slices, we form the supervisory segmentation signal. Detailed experimental procedures and settings are presented in Section 4.4.

(1) $$\begin{array}{c} \;p=\{\begin{array}{c} 1,\;\;\;\;\mathrm{middle}\;\mathrm{slice}\\ \;weight^{d},\;\;\;\mathrm{otherwise} \end{array} \end{array}$$

p represents the confidence of each slice to the source slice, i.e., the middle slice of a certain view dimension, *d* is the distance from slice i to the middle slice, and the distance is the slice size in a specific direction; *weight* is the slice weight set in a certain direction, and the confidence of a slice in the middle of a three-dimensional shape is 1; in all other cases, it follows an exponential relationship with distance.

By incorporating supervisory information from different views, our proposed model can learn more useful information than methods that rely on a single view. In addition, our proposed method can avoid the impact of noise from one view. Moreover, information from different views can enhance the model's ability to recognize different structures. Since different structures exhibit different features in various directions, acquiring information from multiple views improves the model's perception of structural variations. Integrating information from multiple directions enables the model to comprehensively understand the structure and features of medical images, thereby enhancing segmentation accuracy.

### 3.2 Network architecture and data augmentation

**Figure 2:**
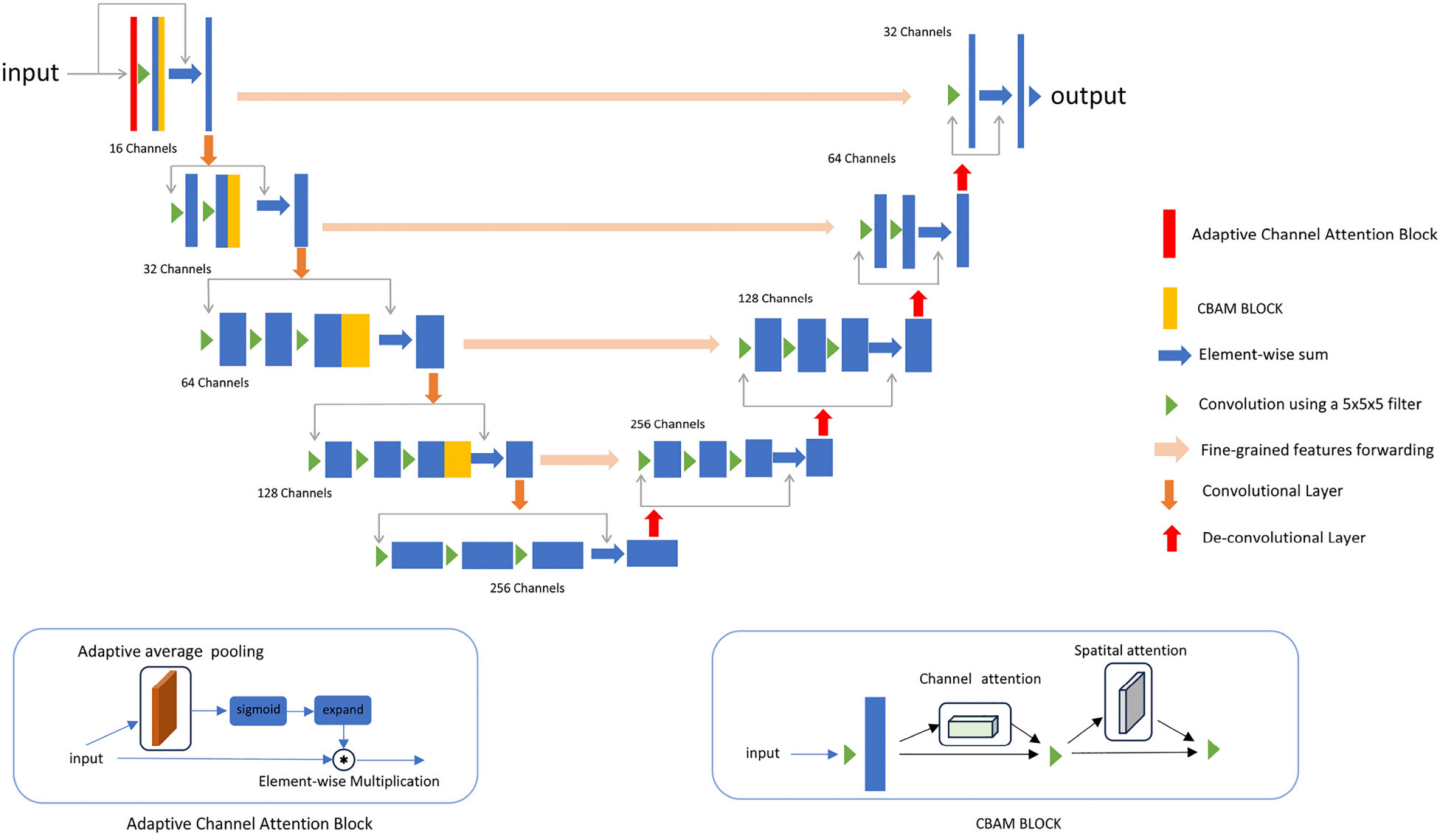
The details of the improved network. An adaptive channel attention block is added to the first layer of the input section, and a CBAM block is added to each downsampling block.

We propose a new 3D segmentation network, as shown in Figure 2, which integrates the CBAM (9) module and adaptive channel attention block (30) into 3D VNet (2). The CBAM attention module, which consists of both channel attention and spatial attention modules, is added to each downsampling layer of the network. The CBAM module made the model focus on the 3D cardiac region from both spatial and channel aspects. In addition, as shown in Figure 2, adaptive channel attention blocks were introduced into the first layer of the input section in the segmentation network. The input data undergoes adaptive average pooling followed by a sigmoid operation, and is then subjected to element-wise convolution with the original data. By incorporating CBAM attention mechanisms and adaptive channel attention block operations, segmentation results in greatly improved boundary delineation of the segmented regions. To investigate the impact of different components in the proposed segmentation network, we propose adding two modules to the VNet: the adaptive channel attention block and the CBAM, aiming to enhance the network performance. These two modules can be added to different positions in the network. For detailed information, please refer to Section 4.4.

We first introduced the CutMix (8) data augmentation method in 3D cardiac segmentation tasks to enhance segmentation performance and robustness. Compared to traditional data augmentation methods, models using CutMix achieved higher segmentation accuracy and better generalization on the validation set.

Let $x\in R^{w\times h\times d}$ represent a training image and y represents its label. The goal is to generate a new training sample ($\tilde{x}$, $\tilde{y}$) by combining two training samples: ($x_{A\;}$, $y_{A}$) and ($x_{B\;}$, $y_{B}$). The generated training sample ($\tilde{x}$, $\tilde{y}$) is used to train the model. The combining operation is defined as follows:

(2) $$\begin{array}{c} \tilde{x}=M\cdot x_{A}+(1-M)\cdot x_{B} \end{array}$$

(3) $$\begin{array}{c} \tilde{y}=M\cdot y_{A}+(1-M)\cdot y_{B} \end{array}$$

Here, $M\in \{0,1{\}}^{W\times H\times D}$ represents a binary mask indicating where to remove and fill in pixels from two images, and “·” denotes element-wise multiplication. Similar to Mixup, the combination ratio *λ* between two data points is sampled from the Beta distribution, Beta(α,α), where *α* = 1, which means *λ* is sampled from the uniform distribution (0,1).

The experimental results showed that the application of CutMix data augmentation improves segmentation accuracy in 3D cardiac medical images by effectively extending the diversity of the dataset.

### 3.3 Loss function

The details of the loss function related to the algorithm are shown in the following formula:

(4) $$\begin{array}{c} L_{total}=L_{sup}+L_{semi}+\lambda \cdot L_{unsup} \end{array}$$

$L_{sup}$ is the loss function of segmentation and ground truth, $L_{semi}$ is the segmentation loss guided by pseudo-labels, applied to both unlabeled data and mixed data, and $L_{unsup}$ is the loss function of uncertainty rectification. $L_{sup}$ and $L_{semi}$ adopt the combination of weighted cross-entropy and weighted dice loss (22). The loss encompasses weighted cross-entropy loss and weighted dice loss:

(5) $$\begin{array}{c} L_{ce}=-\frac{1}{\sum_{i=1}^{H\times W\times D}w_{i}}\overset{H\times W\times D}{\underset{i=1}{\sum }}\;w_{i}y_{i}logp_{i} \end{array}$$

(6) $$\begin{array}{c} L_{dice}=1-\frac{2\times \sum_{i=1}^{H\times W\times D}\;w_{i}y_{i}p_{i}}{\sum_{i=1}^{H\times W\times D}w_{i}(p_{i}^{2}+y_{i}^{2})} \end{array}$$

Where $w_{i}\;$ is ***i***th voxel of weight map $W,\;p_{i}$ denotes the multi-view slice supervision signal, and $y_{i}$ is the pseudo label ($L_{semi}$) or the label $\;(L_{sup})$ of the voxel.

(7) $$\begin{array}{c} L_{unsup}=\frac{1}{S}\frac{\sum_{s=0}^{s-1}\;\sum_{v}\;(p_{s}^{v}-p_{c}^{v}{)}^{2}\cdot w_{s}^{v}}{\sum_{s=0}^{s-1}\;\sum_{v}w_{s}^{v}}\;+\;\frac{1}{S}\overset{s-1}{\underset{s=0}{\sum }}\;||D_{s}|{|}^{2} \end{array}$$

**Figure 3:**
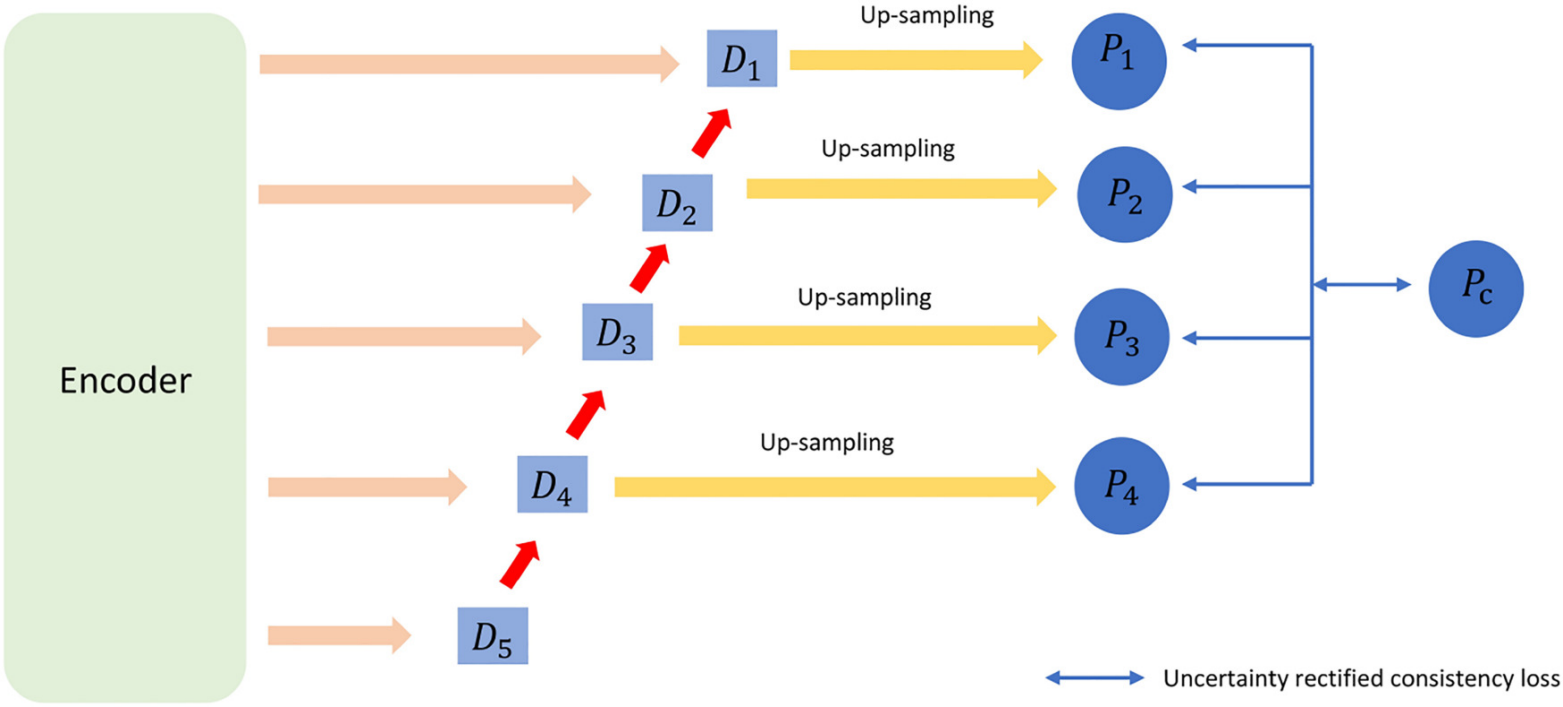
The structure diagram for uncertainty rectification in VNet.

This part consists of two components: uncertainty rectification and uncertainty minimization. For the unlabeled data, we used consistency regularization by enforcing the consistency of multi-scale predictions (21). Thus, $p_{s}^{v}$ and $p_{c}^{v}$ are the corresponding prediction and uncertainty values for voxel v. The structure diagram of uncertainty rectification in VNet is shown as Figure 3.

To be specific, we use the Kullback–Leibler (KL) divergence between the average prediction and the prediction at scales as the uncertainty measurement. $D_{0}$, $D_{1}$, …, $D_{s-1}$ is a set of uncertainty maps where $D_{s}$ corresponds to the uncertainty of $\;p_{s}$.${\;p}_{s}^{j}$ is the *j*th channel of $p_{s}$, and C is the class (i.e., channel) number.

(8) $$\begin{array}{c} D_{S}\approx \overset{C}{\underset{j=0}{\sum }}\;p_{s}^{j}\cdot \log\frac{p_{s}^{j}}{p_{c}^{j}} \end{array}$$

## 4 Experiments

### 4.1 Dataset

In this experiment, we selected two publicly available datasets, the left atrium (LA) dataset and the challenge on endocardial three-dimensional ultrasound segmentation (CETUS) dataset, to validate the proposed method in this paper.

The LA dataset (31) comprises 100 3D gadolinium-enhanced magnetic resonance images obtained using a clinical whole-body MRI scanner, along with comprehensive annotations for the left atrial cavity provided by radiologists. All scans have a consistent isotropic resolution of 0.625 mm^3^ × 0.625 mm^3^ × 0.625 mm^3^, although their dimensions may vary among the scans.

The CETUS dataset (32) includes 3D cardiac ultrasound images from 45 patients, with each patient's images capturing the performance of the left ventricle in both end-diastolic and end-systolic phases. The 45 patients are divided into three groups as follows: 15 healthy patients, 15 patients with a history of myocardial infarction occurring at least 3 months prior to the study, and 15 patients diagnosed with dilated cardiomyopathy.

### 4.2 Implementation details

In the two-directional supervision, we set the position of the supervisory source slice for each data point at the midpoint and used it for supervised training.

For segmentation training, we trained the network using 5% and 10% of the labeled data from the two datasets. Our semi-supervised segmentation model is built based on the mean teacher structure. We employed a sliding window approach with a fixed stride to extract patches. We set the patch size data to 112 × 112 × 80 for the LA dataset and 192 × 192 × 64 for the CETUS dataset. The batch size is 2, with one labeled data point and one unlabeled data point. The training epoch is set to 6,000. During our training process, we employed an stochastic gradient descent (SGD) optimizer with a momentum of 0.9 and weight decay of 0.0001. Our framework is implemented in PyTorch 1.12.0 and utilizes an Nvidia RTX 3090 GPU with 24 GB of memory. All the model training used the VNet. For quantitative evaluation, we employed four metrics: Dice, Jaccard index, average surface distance (ASD), and 95% Hausdorff distance (95HD).

### 4.3 Comparison with state-of-the-art methods

We compared four state-of-the-art semi-supervised segmentation models, including uncertainty–aware mean teacher (UA-MT) (33), shape-aware adversarial network (SASSNet) (34), exploring smoothness and class-separation for semi-supervised medical image segmentation (SS-Net) (35), and mutual consistency network (MC-Net+) (36). In Tables 1–4, we present the results of our method without CBAM and CutMix (Ours) and with CBAM and CutMix (Ours*).

**Table 1:** Comparison of four state-of-the-art semi-supervised methods and our method using the 5% labeled LA dataset as the training dataset.

Method	Scans used		Metrics			
	Labeled	Unlabeled	Dice (%)	Jaccard (%)	95HD (voxel)	ASD (voxel)
UA-MT	4 (5%)	76 (95%)	76.83	65.14	20.86	5.56
SASSNet	4 (5%)	76 (95%)	76.97	63.9	25.08	7.84
SS-Net	4 (5%)	76 (95%)	78.9	66.35	15.2	3.75
MC-Net+	4 (5%)	76 (95%)	80.8	68.86	17.28	4.68
Ours	4 (5%)	76 (95%)	84.8	73.92	14.52	4.03
Ours*	4 (5%)	76 (95%)	**86**.**47**	**76**.**33**	**9**.**68**	**2**.**12**

Ours (without CBAM and CutMix) and Ours* (with CBAM and CutMix).

The best approach is highlighted in bold.

**Table 2:** Comparison of four state-of-the-art semi-supervised methods and our method using the 10% labeled LA dataset as the training dataset.

Method	Scans used		Metrics			
	Labeled	Unlabeled	Dice (%)	Jaccard (%)	95HD (voxel)	ASD (voxel)
UA-MT	8 (10%)	72 (90%)	86.2	76	13.87	3.46
SASSNet	8 (10%)	72 (90%)	84.53	73.93	12.05	3.32
SS-Net	8 (10%)	72 (90%)	85.57	75.63	10.05	2.37
MC-Net+	8 (10%)	72 (90%)	86.32	76.2	14.17	3.77
Ours	8 (10%)	72 (90%)	86.96	77.11	12.9	3.51
Ours*	8 (10%)	72 (90%)	**87**.**83**	**78**.**48**	**7**.**78**	**2**.**18**

Ours (without CBAM and CutMix) and Ours* (with CBAM and CutMix).

The best approach is highlighted in bold.

**Table 3:** Comparison of four state-of-the-art semi-supervised methods and our method using the 5% labeled CETUS dataset as the training dataset.

Method	Scans used		Metrics			
	Labeled	Unlabeled	Dice (%)	Jaccard (%)	95HD (voxel)	ASD (voxel)
UA-MT	2 (5%)	38 (95%)	85.12	74.36	24.41	5.97
SASSNet	2 (5%)	38 (95%)	85.27	74.79	21.15	5.59
SS-Net	2 (5%)	38 (95%)	84.78	73.96	19.71	6.04
MC-Net+	2 (5%)	38 (95%)	86.44	76.38	18.36	**5**.**3**
Ours	2 (5%)	38 (95%)	86.56	76.78	15.87	5.58
Ours*	2 (5%)	38 (95%)	**87**.**71**	**78**.**61**	**14**.**82**	**5**.**33**

Ours (without CBAM and CutMix) and Ours* (with CBAM and CutMix).

The best approach is highlighted in bold.

**Table 4:** Comparison of four state-of-the-art semi-supervised methods and our method using the 10% labeled CETUS dataset as the training dataset.

Method	Scans used		Metrics			
	Labeled	Unlabeled	Dice (%)	Jaccard (%)	95HD (voxel)	ASD (voxel)
UA-MT	4 (10%)	36 (90%)	86.23	76.03	16.91	5.44
SASSNet	4 (10%)	36 (90%)	86.3	76.3	**13**.**02**	4.56
SS-Net	4 (10%)	36 (90%)	85.65	75.26	13.78	5.02
MC-Net+	4 (10%)	36 (90%)	86.94	77.25	17.43	5.21
Ours	4 (10%)	36 (90%)	87.8	78.63	16.65	4.91
Ours*	4 (10%)	36 (90%)	**88**.**49**	**79**.**69**	13.34	**3**.**87**

Ours (without CBAM and CutMix) and Ours* (with CBAM and CutMix).

The best approach is highlighted in bold.

**Figure 4:**
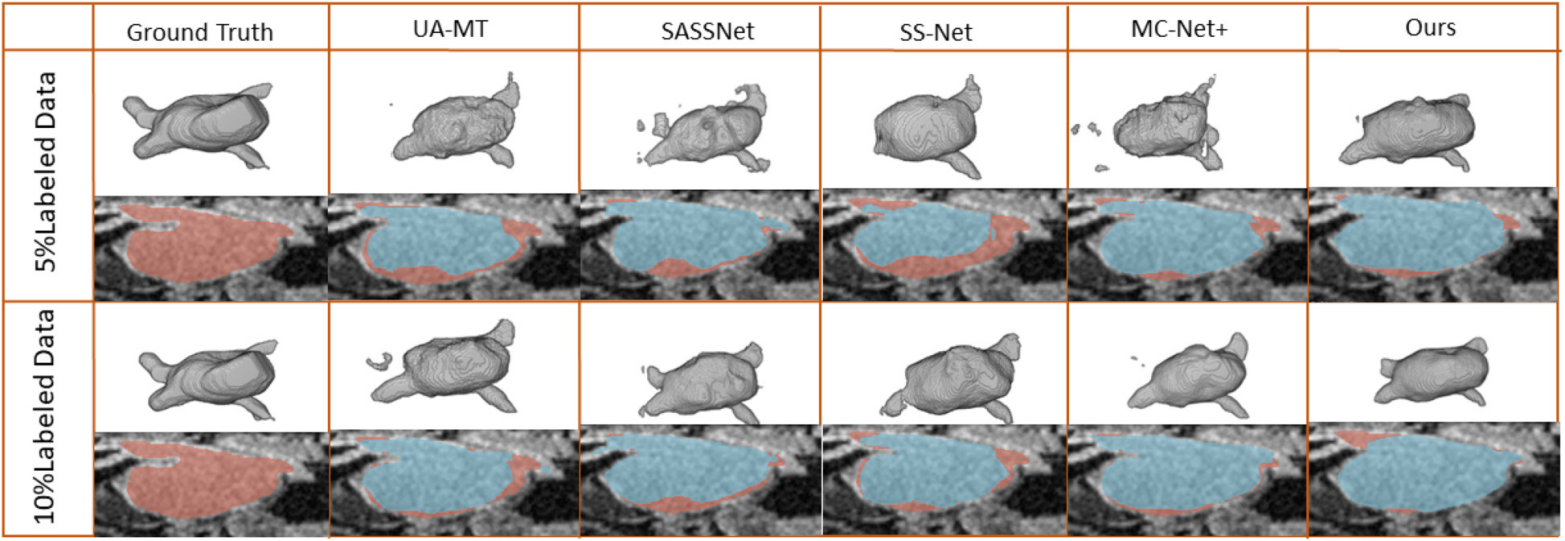
2D and 3D visualization and other methods on the LA dataset with 5% and 10% labeled data. The red areas represent the ground truth, while the blue areas represent predictions.

We trained on the LA dataset using 5% and 10% of the labeled data, and the results showed that the effect was excellent on small sample datasets. Table 1 shows the result of our model and four SOTA models using a 5% labeled LA dataset as a training dataset. The experimental results show that our model achieved a significant improvement across four evaluation metrics. Our model, with only 5% labeled data, achieved a Dice score of 86.47%, Jaccard index of 76.33%, 95HD of 9.68, and ASD of 2.12, outperforming the other four SOTA models. In terms of the Dice segmentation metric, our method outperformed UA-MT by 9.64%, SASSNet by 9.5%, SS-Net by 7.57%, and MC-Net+ by 5.67%. For the 95HD evaluation metric, our method showed a reduction of 11.18 compared to UA-MT, 15.4 compared to SASSNet, 5.52 compared to SS-Net, and 7.6 compared to MC-Net+. In addition, as shown in Figure 4, our model achieved the best segmentation results compared to other models. The segmentation results did not exhibit over-segmentation, and better segmentation was achieved in the pulmonary vein.

**Figure 5:**
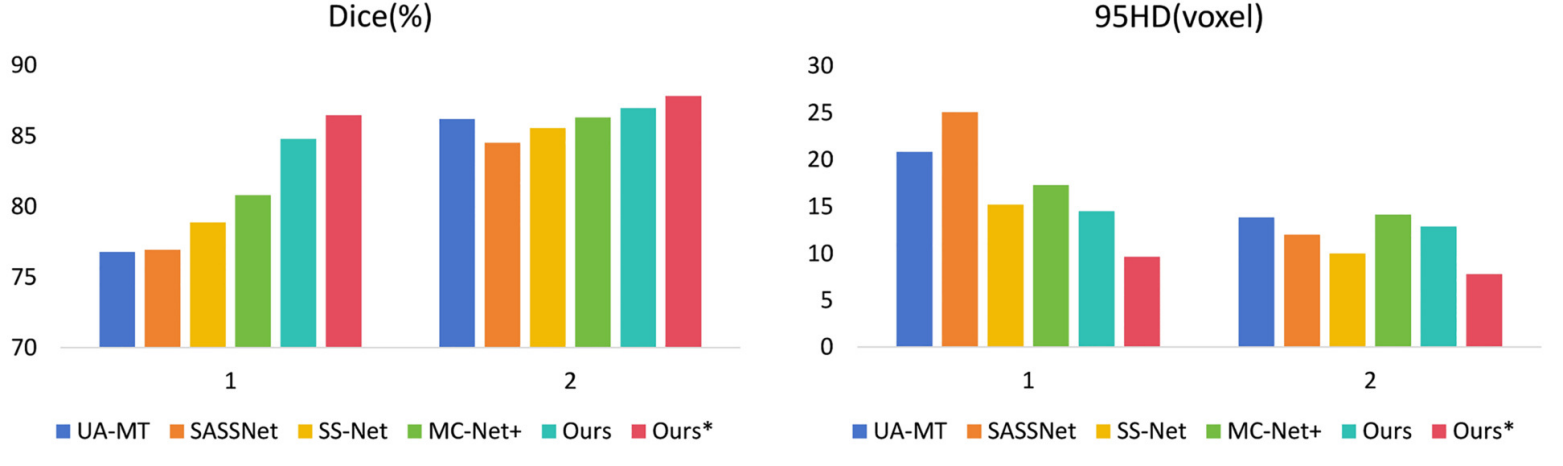
Comparison of our method with four state-of-the-art semi-supervised methods on the LA dataset. (1 represents 5% labeled data and 2 represents 10% labeled data).

In Table 2, with 10% labeled data, we also achieved good results. As the labeled dataset size increased, the segmentation results of all models were improved. Our model achieved a Dice score of 87.83%, Jaccard index of 78.48%, 95HD of 7.78, and ASD of 2.18, outperforming the other four SOTA models. In Figure 4, it can be observed that the segmentation results of the other models had many discontinuous parts. Moreover, in the central positions, our model's results are the closest to the ground truth. Figure 5 shows the comparison between our method and four state-of-the-art semi-supervised learning methods on the LA dataset with 5% labeled data and 10% labeled data.

**Figure 6:**
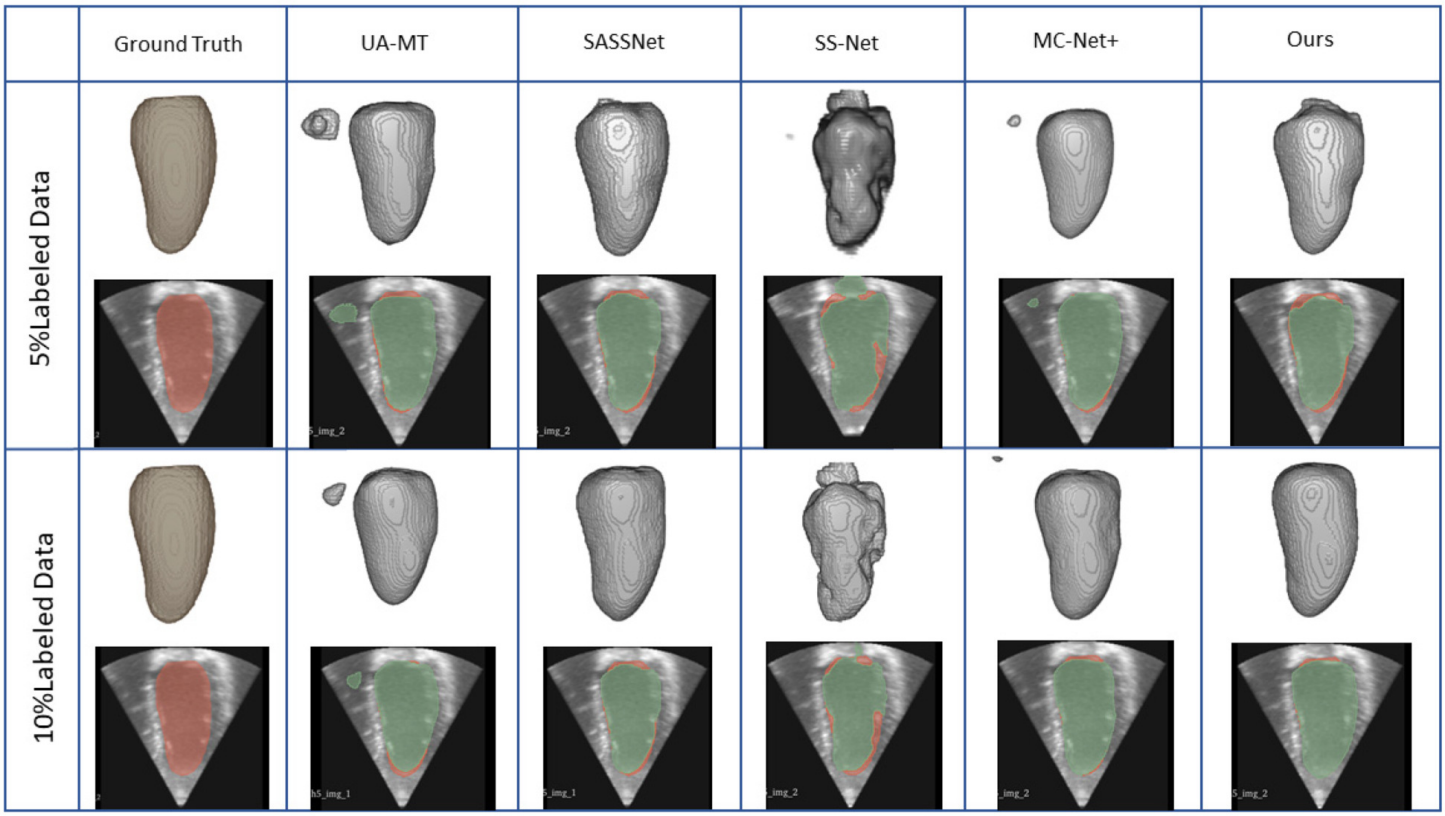
The positions of the CBAM block and the adaptive channel attention block in our proposed enhanced VNet across experimental methods 1, 2, 3, and 4.

**Figure 7:**
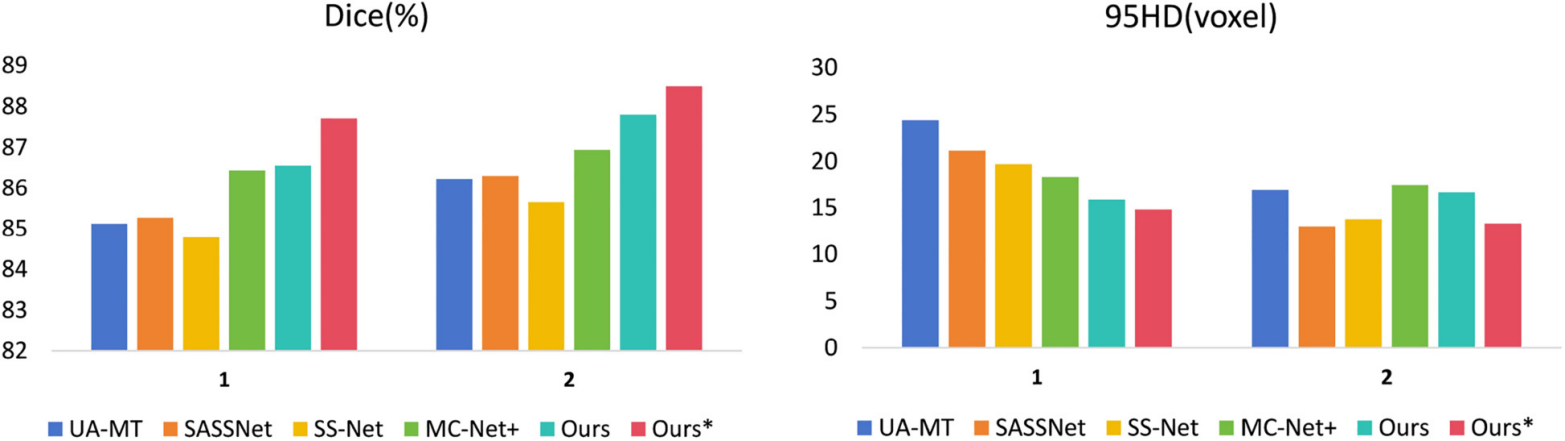
The 3D segmentation results and the different slice supervision signals on the LA dataset.

We trained each model with the CETUS dataset using 5% and 10% of the labeled data. Table 3 shows the results of our model and the other state-of-the-art methods with the 5% labeled CETUS dataset. Our model achieved the best segmentation performance. Our model achieved good results on the 5% labeled dataset, with a Dice score of 87.71%, Jaccard index of 78.61%, 95HD of 14.82, and ASD of 5.33. In terms of the Dice segmentation metric, our method outperformed UA-MT by 2.59%, SASSNet by 2.44%, SS-Net by 2.93%, and MC-Net+ by 1.27%. Figure 6 shows that our method achieved better results than other methods on both core and edge regions, with our model's segmentation results being more complete and the edges closer to the ground truth compared to other models. In Table 4, on the 10% CETUS dataset, the experimental results indicated that our performance was better than other state-of-the-art models in terms of both Dice and Jaccard indices. The Dice score, Jaccard index, 95HD, and ASD were 88.49%, 79.69%, 13.34, and 3.87 respectively. Figure 7 shows the comparison between our method and four state-of-the-art semi-supervised learning methods on the CETUS dataset with 5% labeled data and 10% labeled data.

### 4.4 The impact of our proposed methods on the results

#### 4.4.1 Different components of the CBAP-VNet segmentation network

An adaptive channel attention block can be added to the downsampling layers of the input part in the VNet segmentation network, and the CBAM module can be incorporated into both the upsampling and downsampling layers of the VNet. We compared the segmentation accuracy with different numbers and positions of the CBAM modules. We also evaluated the importance of the adaptive channel attention block. We validated the performance of the model using 5% of the labeled dataset from the CETUS and LA datasets.

**Figure 8:**
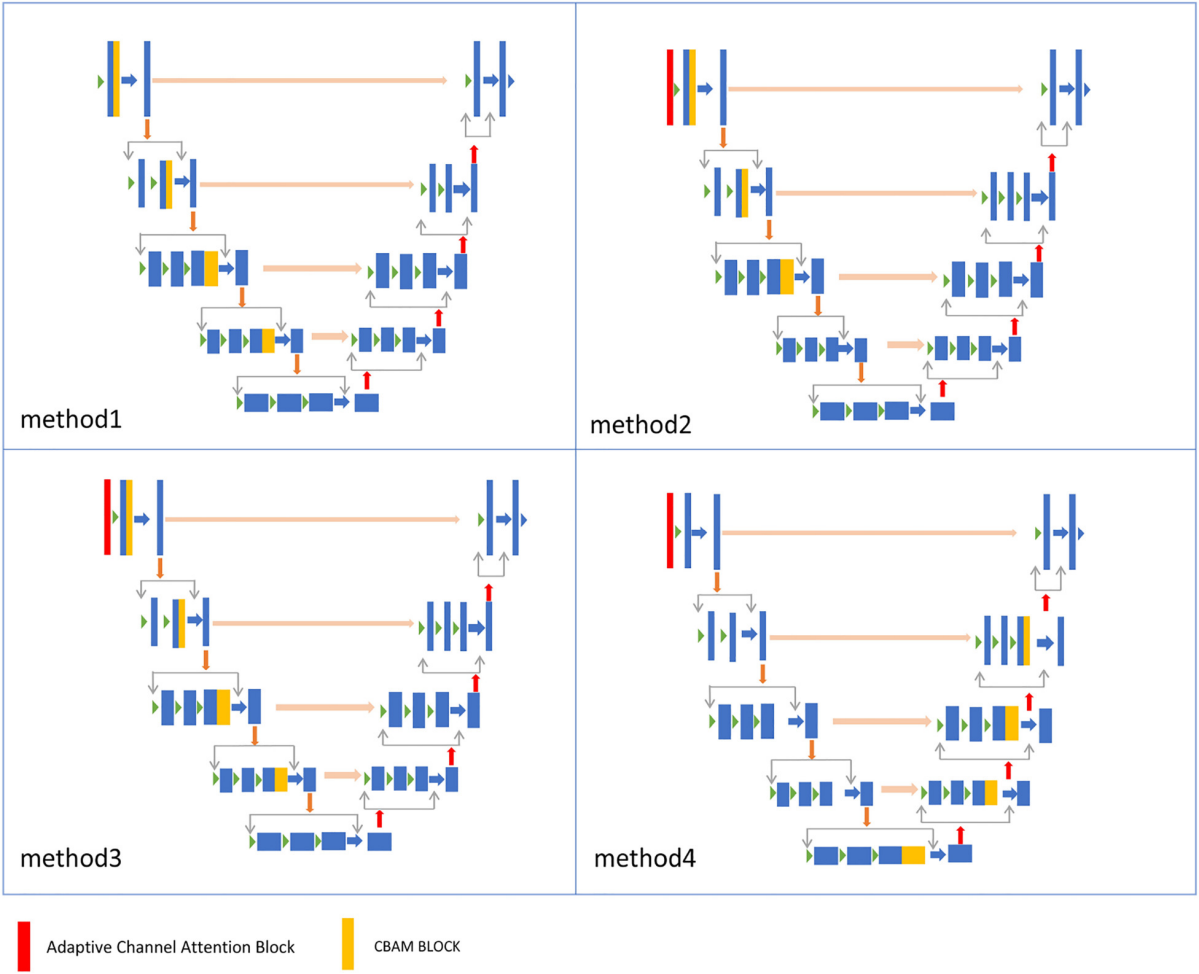
The 3D segmentation results and the different slice supervision signals on the CETUS dataset.

In Figure 8, Method 1 adds the CBAM module to four downsampling layers. Method 2 adds the CBAM module to three downsampling layers and incorporates an adaptive channel attention block in the input part. Method 3 adds the CBAM module to four downsampling layers and incorporates an adaptive channel attention block in the input part. Method 4 adds the CBAM module to four upsampling layers and incorporates an adaptive channel attention block in the input part. Method 5 is the original VNet without adding any modules. Method 3 demonstrated the best performance on both the LA and CETUS datasets, as shown in Tables 5, 6, with a Dice score of 86.47% and 87.71%, Jaccard index of 76.33% and 78.61%, 95HD of 9.68 and 14.82, and ASD of 2.12 and 5.33, respectively. The experimental results indicated that method 3, adding an adaptive channel attention block to the downsampling layers of the input in the VNet 3D segmentation network and incorporating CBAM modules into all four network layers, yields the best segmentation performance.

**Table 5:** The effect of different components of the proposed method on the performance metrics with the 5% labeled CETUS dataset.

Method	Adaptive channel attention block	CBAM3	CBAM4	CBAM4（up）	Dice (%)	Jaccard (%)	95HD (voxel)	ASD(voxel)
1			√		84.75	73.8	10.7	2.69
2	√	√			83.27	71.64	13.2	3.8
3	√		√		**86**.**47**	**76**.**33**	**9**.**68**	**2**.**12**
4	√			√	81.72	69.73	15.48	4.36
5					85.24	74.56	10.42	2.85

The bold values are the best results in the comparative experiment.

**Table 6:** The effect of the different components of the proposed method on the performance metrics with the 5% labeled CETUS dataset.

Method	Adaptive channel attention block	CBAM3	CBAM4	CBAM4 (up)	Dice (%)	Jaccard (%)	95HD (voxel)	ASD (voxel)
1			√		87.22	77.92	16.44	4.27
2	√	√			87.24	16.44	13.2	4.92
3	√		√		**87**.**71**	**78**.**61**	**14**.**82**	**5**.**33**
4	√			√	80.65	68.02	46.69	11.9
5					86.84	77.33	16.87	5.65

The bold values are the best results in the comparative experiment.

#### 4.4.2 The slice selection settings

We experimented with the slice selection settings for multi-view supervision and evaluated their impact on segmentation accuracy. The selection of slices in two directions serves as supervision for handling segmentation details. To validate the model's effectiveness, we chose slices from the central region in both directions, slices from the edges, and multiple slices. These selections were examined for their influence on the final experimental results. To validate the experimental outcomes, we conducted experiments on a 5% subset of the LA and CETUS datasets.

**Figure 9:**
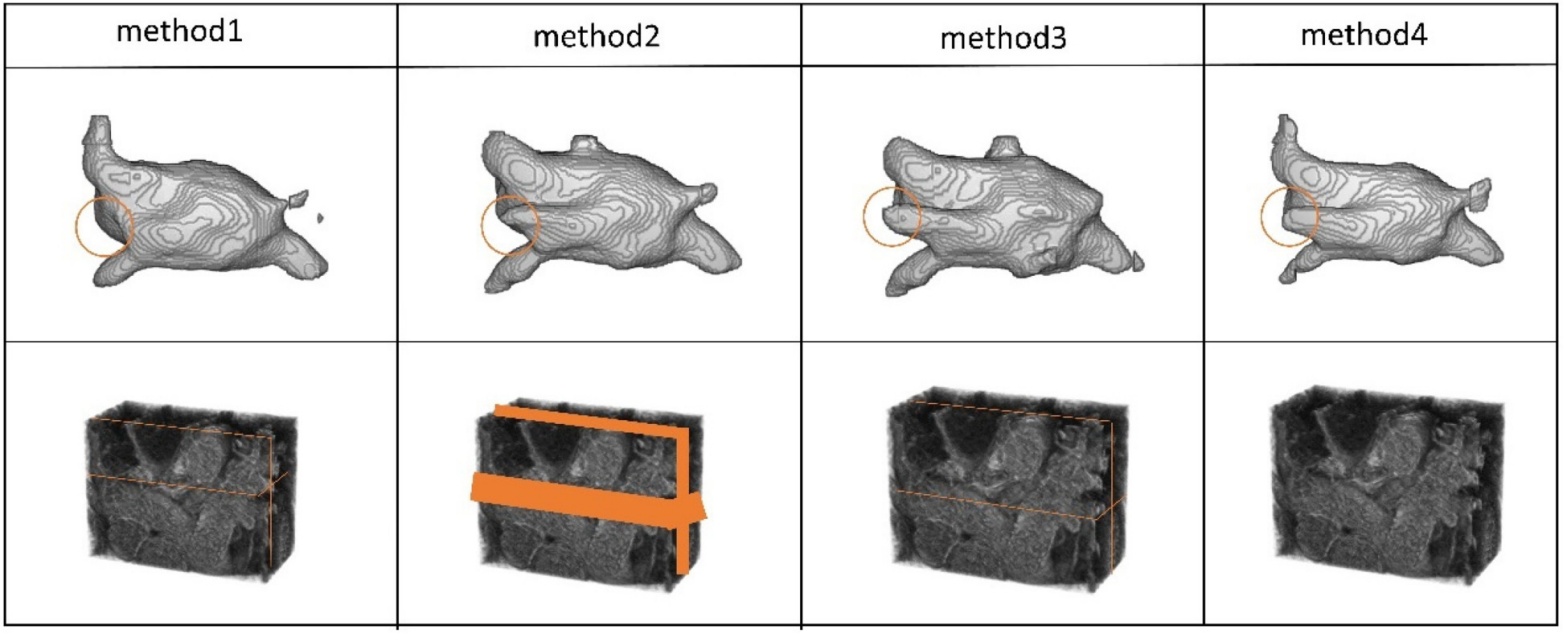
The 3D segmentation results and the different slice supervision signals on the LA dataset.

We selected slices at three positions to validate the effectiveness of the multi-view. Method 1: The slices from the edge of the data. Method 2: The slices were from the continuous area in the middle of the data. Method 3: The slices were from the middle of the data. Method 4 refers to the approach without adding slice supervision signals. In Table 7 and Figure 9, the results of our experiments on the LA dataset, which contains only 5% labeled data, are presented. For Method 1, the results were as follows: Dice coefficient was 84.51%, Jaccard index was 73.38%, 95HD was 11.48, and ASD was 2.98. It can be observed that the edge regions in the 3D segmentation results were missing. For Method 2, the results were: Dice coefficient was 86.2%, Jaccard index was 75.92%, 95HD was 9.36, and ASD was 2.57. Although there was some improvement in the segmentation of the edge regions, the overall performance remained suboptimal. In Method 3, the segmentation results were the best, with the best values for the four performance metrics: Dice coefficient was 86.47%, Jaccard index was 76.33%, 95HD was 9.68, and ASD was 2.12. Regarding Method 4, the results were: Dice coefficient was 84.6%, Jaccard index was 73.59%, 95HD was 10.76, and ASD was 2.94.

**Table 7:** Results of slice selection experiments on the 5% labeled LA dataset.

Method	Dice	Jaccard	95HD	ASD
1	84.51	73.38	11.48	2.98
2	86.2	75.92	**9**.**36**	2.57
3	**86**.**47**	**76**.**33**	9.68	**2**.**12**
4	84.6	73.59	10.76	2.94

The bold values are the best results in the comparative experiment.

**Figure 10:**
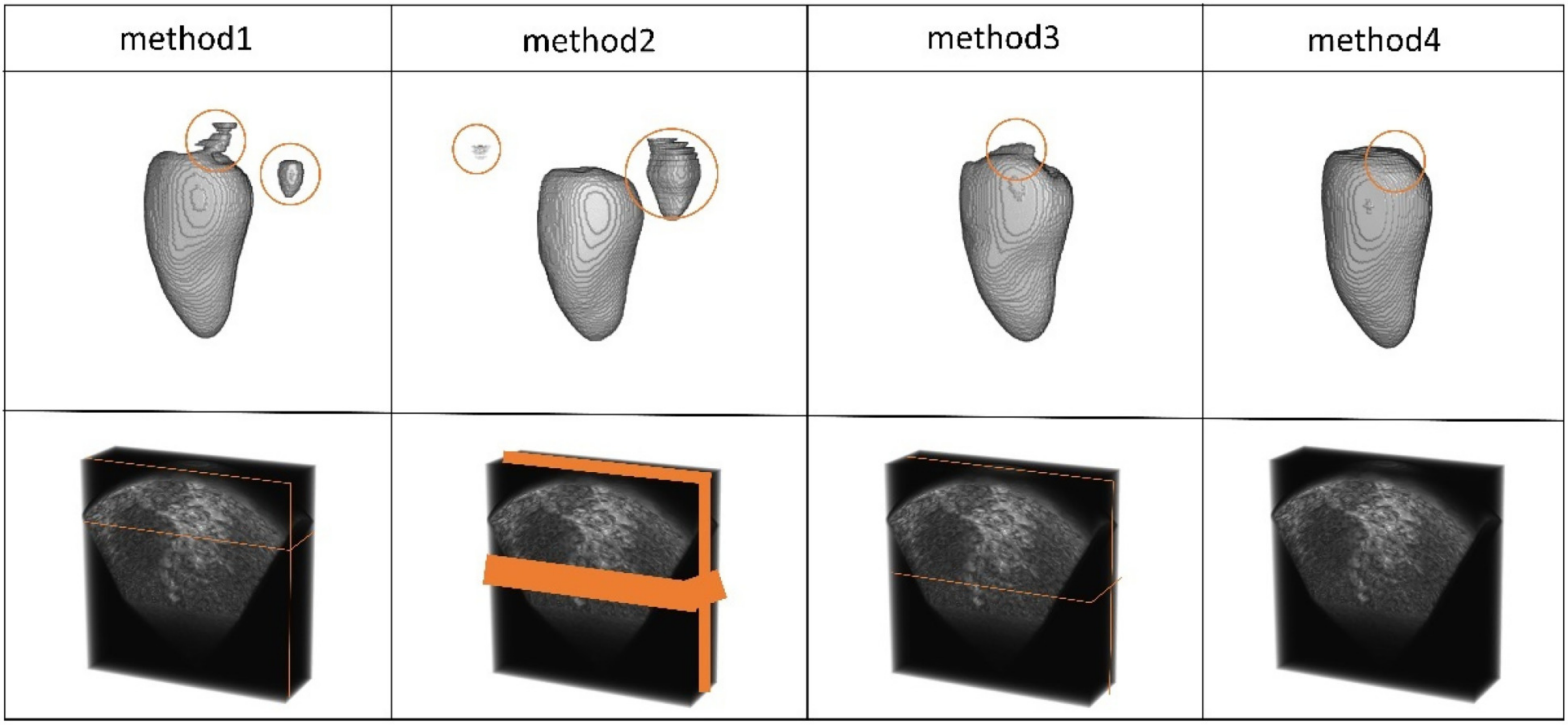
The 3D segmentation results and the different slice supervision signals on the CETUS dataset.

In Table 8 and Figure 10, the results of our experiments on the CETUS dataset, which contains only 5% labeled data, are presented. For Method 1, the results were as follows: Dice coefficient was 86.97%, Jaccard index was 77.25%, 95HD was 18.64, and ASD was 5.14. In the 3D segmentation results, although the edge segmentation was acceptable, many parts were unrelated to the ground truth labels. For Method 2, the results were as follows: Dice coefficient was 86.47%, Jaccard index was 76.58%, 95HD was 20.63, and ASD was 6.49. There were significant parts that were unrelated to the ground truth labels. In Method 3, the segmentation results were the best, with all four performance metrics reaching their highest values: Dice coefficient was 87.71%, Jaccard index was 78.61%, 95HD was 14.82, and ASD was 5.33. Figures 9, 10 indicate that better segmentation results were achieved from slices in the middle from both views. Regarding Method 4, the results were as follows: Dice coefficient was 86.41%, Jaccard index was 76.59%, 95HD was 19.56, and ASD was 5.67.

**Table 8:** Results of slice selection experiments on the 5% labeled CETUS dataset.

Method	Dice	Jaccard	95HD	ASD
1	86.97	77.25	18.64	5.14
2	86.47	76.58	20.63	6.49
3	**87**.**71**	**78**.**61**	**14**.**82**	**5**.**33**
4	86.41	76.59	19.56	5.67

The bold values are the best results in the comparative experiment.

### 4.5 Ablation study

In our framework, we conducted ablation experiments to evaluate the effectiveness of the CutMix module, the multi-view guide module, and the CBAP-VNet model. The following are the results of our ablation experiments conducted on the 5% labeled LA dataset and the 5% labeled CETUS dataset. To better understand and evaluate the components of our method, we sequentially introduced each setting of our experiment: (1) the CutMix module, (2) the multi-view guide module, and (3) CBAP-VNet. The results are shown in Tables 9, 10.

**Table 9:** Results of ablation experiments on the 5% labeled LA dataset.

Method	Mix	Multi-view	CBAP-VNet	Dice	Jaccard	95HD	ASD
1				83.87	72.58	11.83	2.92
2	√			84.65	73.67	11.41	2.55
3	√	√		85.24	74.56	10.42	2.85
4	√	√	√	**86**.**47**	**76**.**33**	**9**.**68**	**2**.**12**

The bold values are the best results in the comparative experiment.

**Table 10:** Results of ablation experiments on the 5% labeled CETUS dataset.

Method	Mix	Multi-view	CBAP-VNet	Dice	Jaccard	95HD	ASD
1				84.44	73.59	22.19	6.46
2	√			85.22	74.68	21.08	6.5
3	√	√		86.84	77.33	16.87	5.65
4	√	√	√	**87**.**71**	**78**.**61**	**14**.**82**	**5**.**33**

The bold values are the best results in the comparative experiment.

The experimental results show that segmentation performance steadily improved across the four metrics throughout the entire training process as the three modules were sequentially combined. In Table 9, when all three modules were used on the LA dataset, the Dice coefficient reached 86.47%, the Jaccard index was 76.33%, the 95HD was 9.68, and the ASD was 2.12. Table 10, presents the results when all three modules were used on the CETUS dataset. The result showed that the Dice coefficient was 87.71%, the Jaccard index was 78.61%, 95HD was 14.82, and ASD was 5.33. The ablation experiments demonstrate that each module had a significant enhancing effect on the segmentation results of the two datasets.

## 5 Conclusions

This paper proposes a novel framework for 3D cardiac image segmentation based on the mean teacher network structure. We employed multi-view supervision to obtain supervision slices from both coronal and transverse views. In this way, the proposed model gathered more comprehensive supervision information and paid more attention to core and edge regions. In addition, we improved the segmentation network by incorporating CBAM and adaptive channel attention into the VNet architecture to capture better features for segmentation. Due to the limited size of medical image datasets, we applied the CutMix data augmentation technique to increase the dataset size. In this way, the proposed method achieved better segmentation performance. The experimental results demonstrated the feasibility and effectiveness of our proposed model.

## Data Availability

The original contributions presented in the study are included in the article/Supplementary Material, further inquiries can be directed to the corresponding author.
